# 

**Published:** 1895-10

**Authors:** 


					﻿Southern Medical Record.
A Monthly Jourr.>f Medicine and Surgery.
Vol. XXV. ATLANTA, GA.. OCTOBER, 1895.	No. loM
BERNARD WOLFF, M. D., Editor and Proprietor.
Associate Editors :
A W. GRIGGS, M. D.
j. mcfadden gaston, m. d.
WILLIS F. WESTMORELAND, M.D.
LOUIS H. JONES, M. D.
Entered at the Post-office at Atlanta, Ga., as Second-Class Matter.
The Foote & Davies Co., Atlanta, Ga.
				

